# Participation of 5-HT and AT_1_ Receptors within the Rostral Ventrolateral Medulla in the Maintenance of Hypertension in the Goldblatt 1 Kidney-1 Clip Model

**DOI:** 10.1155/2014/723939

**Published:** 2014-01-21

**Authors:** Cássia T. Bergamaschi, Nyam F. Silva, Jose G. Pires, Ruy R. Campos, Henrique A. Futuro Neto

**Affiliations:** ^1^Federal University of São Paulo, School of Medicine, Rua Botucatu 862, 04023-060 São Paulo, SP, Brazil; ^2^Federal University of Espirito Santo, 29042-751 Vitória, ES, Brazil; ^3^UNIVIX Medical School, 29075-080 Vitória, ES, Brazil; ^4^UNESC Medical School, 29703-900 Colatina, ES, Brazil; ^5^EMESCAM Medical School, 29045-402 Vitória, ES, Brazil

## Abstract

The hypothesis that changes in neurotransmission within the rostral ventrolateral medulla (RVLM) are important to maintain the high blood pressure (BP) was tested in Goldblatt one kidney-one clip hypertension model (1K-1C). Male Wistar rats were anesthetized (urethane 1.2 g/kg, i.v.), and the effects of bilateral microinjections into the RVLM of the following drugs were measured in 1K-1C or control groups: glutamate (0.1 mol/L, 100 nL) and its antagonist kynurenic acid (0.02 mol/L, 100 nL), the angiotensin AT_1_ receptor antagonist candesartan (0.01 mol/L, 100 nL), and the nonselective 5-HT receptor antagonist methiothepin (0.06 mol/L, 100 nL). Experiments in 1K-1C rats were performed 6 weeks after surgery. In anesthetized rats glutamate response was larger in hypertensive than in normotensive rats (H: Δ67 ± 6.5; N: Δ43 ± 3.54 mmHg). In contrast, kynurenic acid microinjection into the RVLM did not cause any change in BP in either group. The blockade of either AT_1_ or 5-HT receptors within the RVLM decreased BP only in 1K-1C rats. A largest depressor response was caused by 5-HT receptor blockade. The data suggest that 5-HT and AT_1_ receptors act tonically to drive RVLM in 1K-1C rats, and these actions within RVLM contribute to the pathogenesis of this model of hypertension.

## 1. Introduction

The importance of sympathetic nervous system activation in the pathogenesis of hypertension has been demonstrated, and the therapeutic value of sympathetic nervous inhibition in hypertensive patients is already evident and has been widely studied [1]. Ongoing activity of premotor rostral ventrolateral medulla (RVLM) neurons is responsible for the tonic generation of sympathetic vasomotor tone; inhibition of RVLM neurons causes a large decrease in both arterial blood pressure (BP) and sympathetic nervous system activity, while stimulation of this medullary region increases sympathetic vasomotor outflow and BP [2, 3]. Therefore, changes in the local neurotransmission within the RVLM can be a mechanism involved in the sympathetic activation in hypertension.

Previous studies demonstrated that microinjection into the RVLM of excitatory amino acid (EAA) receptor antagonists has no effect on basal level of BP [4]. This fact has been interpreted as suggesting that the ongoing RVLM activity is not dependent on EAA inputs to the RVLM. However, we showed previously that, in Goldblatt 2-kidneys, one-clip (2K1C) model, microinjection of kynurenic acid, a broad spectrum EAA receptor antagonist, into the RVLM, reduced BP to the same extent as autonomic blockade [5]. Similar results were later shown in SHR and in Dahl-salt sensitive rats [6, 7]. On basis of these results, it was proposed that tonic actions of glutamatergic inputs to the RVLM are involved in the pathogenesis of experimental hypertension. However, it is not well known if the glutamatergic tonic action on RVLM is specific for those models or if it appears in other experimental models of hypertension, such as the Goldblatt 1-kidney, one-clip (1K-1C) model. Part of the present study was undertaken to unravel this question.

Although there is a general agreement on the importance of the renin-angiotensin system for increased BP in the renovascular hypertension, several findings suggest that the hypertensive response to angiotensin II (Ang II) is neurogenically mediated [7]. In this case, a possible mechanism responsible for altering the RVLM neurotransmission in the renovascular hypertension is the increase of circulating Ang II, which, for instance, can indirectly drive glutamatergic inputs to RVLM through the area postrema [8]. Therefore, the possibility that changes in the action of Ang II within the RVLM contribute to the renovascular hypertension cannot be excluded. For instance, tonic actions of AT_1_ Ang II receptors into the RVLM contribute to maintain the salt-dependent hypertension [9], and an increase in sympathetic activity in response to activation of paraventricular nucleus is mediated by AT_1_ receptors within the RVLM [10]. The present study also tested the hypothesis that activation of AT_1_ receptors within the RVLM contributes to the increase in BP in the 1K-1C model.

Finally, a possible change in 5-hydroxytryptamine (5-HT) receptors within the RVLM may also be involved in the maintenance of high BP in renovascular hypertension, since it is well known that activation of 5-HT_1/2_ receptors in the RVLM cause sympathoexcitation and a rise in BP [11]. Therefore, activation of 5-HT receptors in the RVLM can increase BP and sympathetic activity in renovascular hypertension. This hypothesis was also tested in the present study. On the basis of the hypothesis that changes in neurotransmission within the RVLM in 1K-1C model contribute to maintain high BP, the present study begins to test in the first series of experiments, in renovascular hypertensive rats, the phasic and tonic actions of glutamate into the RVLM, by injecting glutamate and its antagonist (kynurenic acid) into the region. In the second and third series of experiments, we, respectively, examined the effects of blocking AT_1_ or 5-HT receptors within the RVLM.

## 2. Methods

Experiments were performed in male Wistar rats (*n* = 22, 250–300 g). All animal procedures were conducted according to the “Guidelines for Ethical Care of Experimental Animals” and were approved by the Institutional Ethics Committee of the School of Medicine federal University of São Paulo. To obtain hypertensive animals, the left renal artery was partially obstructed with a silver clip of 0.2 mm width and the contralateral kidney was removed (Goldblatt hypertension 1-kidney 1-clip model). The animals were submitted to the experimental procedures 6 weeks after the surgery. After this period, rats were anesthetized with ketamine plus xylazine (40 and 20 mg/kg, i.p., resp.) and instrumented with femoral venous and arterial catheters made from PE-50 and PE-10 tubing, filled with heparinized saline, for drug injection and BP recording. In the experimental day, rats were anesthetized with urethane (1.2 g/kg, i.v.), given a tracheotomy, and ventilated artificially with O_2_-enriched air. Rectal temperature was maintained at 37°C by a means of a servocontrolled electric blanket (Harvard). An adequate depth of anesthesia was monitored by observing blood pressure, heart rate, and the corneal and paw-pinch reflexes, and if needed, an additional administration of anesthetic was performed (5% of initial dose).

Animals were placed in a stereotaxic frame and an occipital craniotomy was performed to expose the dorsal surface of the brain stem and cerebellum. The dura mater was opened and retracted exposing the Obex, whose vertex was taken as a landmark for the stereotaxic coordinates. The RVLM was located 3 mm rostral to the Obex, 1.7 to 1.8 mm lateral to midline, and 3 mm deep to the dorsal medullary surface. Bilateral microinjections were made with the use of glass micropipettes with tip diameter of 10–20 *μ*m connected to a pressure injector; the volume injected was measured by determining the displacement of the meniscus in the pipette with respect to a horizontal grid viewed through an operating microscope. First, the injection was made on one RVLM side and then into the contralateral side. There was a delay of approximately 10 s between the injections. In all experiments, glutamate was first microinjected into the RVLM to verify that the coordinates had placed the pipette into a functional pressor RVLM site. Microinjections consisted of glutamate (0.1 mol/L; Sigma), the glutamate antagonist-kynurenic acid (0.02 mol/L; Sigma), Ang II AT_1_ antagonist-candesartan (0.01 mol/L; Astra), and the broad spectrum 5-HT receptor antagonist-methiothepin (0.06 mol/L; Research Biochemical Inc.), in a volume of 100 nL. The pH of the solutions was adjusted to 7.4.

At the end of the experiment, 100 nL of Evans blue dye was injected into the site. The animals were killed by an overdose of urethane and the brain stem was then removed and fixed by immersion for at least 72 h in 4% paraformaldehyde solution. Transverse 40 *μ*m frozen sections were cut and mounted. When the dye was bilaterally deposited ventral to nucleus ambiguous (NA) and lateral to inferior olivary nucleus, this was considered a positive histology.

All values are expressed as means ± SEM. The statistical significance of changes in mean arterial pressure (MAP) or heart rate (HR) after microinjections was determined within each group by Student's paired *t*-test. Differences between groups were assessed by one-way ANOVA followed by the Student-Newman-Keuls test. A value of *P* < .05 was considered significant.

## 3. Results

After 6 weeks of surgery, the rats submitted to the Goldblatt 1-kidney 1-clip procedure developed a significant increase in MAP (176 ± 4.5 mmHg, *P* < .05, *n* = 12), compared to normotensive rats (105 ± 4 mmHg, *n* = 10). There was no difference between groups in relation to heart rate or body weight. Before testing the effects of different microinjections into the RVLM, the functional pressor sites were identified by bilateral glutamate microinjection into the region (0.1 mol/L, 100 nL).

### 3.1. Effects of L-Glutamate and Its Antagonist Microinjected into the RVLM of Renovascular Hypertensive Rats

**Figure 1:**
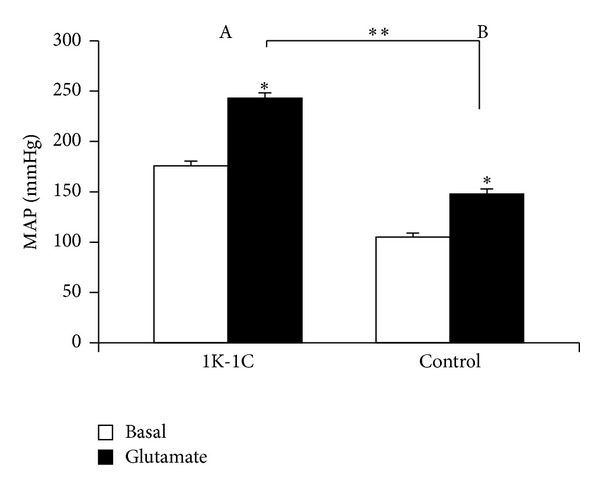
Values of mean arterial pressure (MAP) reached after bilateral microinjection of glutamate (0.1 mol/L, 100 nL) into the RVLM in 1K-1C (*n* = 12) or control rats (*n* = 10). *P < .05 in relation to basal level and **P < .05 comparison between groups.

Bilateral microinjections of glutamate into the RVLM of hypertensive rats caused a significant increase in MAP (from 176 ± 4.5 to 243 ± 5.5 mmHg, *P* < .05, *n* = 12) as shown in Figure 1(A)). with no significant change in HR (from 418 ± 14 to 420 ± 20 bpm). In normotensive rats, a significant increase in MAP was also observed (from 105 ± 4 to 148 ± 5 mmHg, *P* < .05, *n* = 10; Figure 1(B)), without significant changes in HR (from 375 ± 18 to 399 ± 20 bpm). The response to glutamate into the RVLM in hypertensive rats was greater than that in normotensive ones, with a significant difference between groups when compared the absolute increase in MAP (H: Δ + 67 ± 6.5 and N: Δ + 43 ± 3.4 mm Hg, *P* < .05). However, no difference was found in the percentage of variation between groups (H: 39% and N: 40%).

However, when glutamatergic synapses within the RVLM were blocked by bilateral microinjections of kynurenic acid (0.02 mol/L, 100 nL), a broad-spectrum ionotropic glutamate receptor antagonist, in hypertensive animals, no significant changes in MAP (from 174 ± 7 to 185 ± 10, *n* = 6) or in HR (from 413 ± 34 to 426 ± 24 bpm) were achieved. In normotensive rats, the same microinjection did not promote any significant changes in MAP (from 108 ± 4 to 119 ± 6 mmHg, *n* = 5) or HR (from 365 ± 11 to 378 ± 21 bpm).

### 3.2. Effects of Ang II AT_1_ Receptor Blockade in the RVLM of Goldblatt Hypertensive Rats

**Figure 2:**
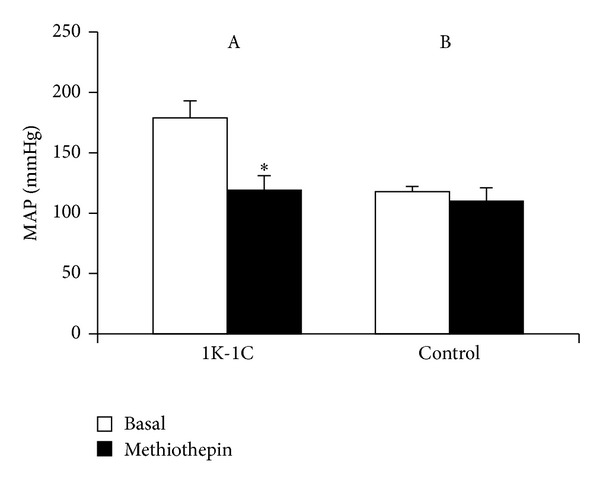
Values of mean arterial pressure (MAP) reached after bilateral microinjection of candesartan (0.01 mol/L, 100 nL) into the RVLM in 1K-1C (*n* = 5) or control rats (*n* = 5). *P < .05 in relation to basal level.

In this series of experiments we tested whether the elevated BP in Goldblatt one-kidney one-clip model is supported by activation of AT_1_ Ang II receptors within the RVLM. To address this issue, 1 nmol of candesartan was bilaterally injected into the RVLM of renovascular hypertensive or control rats. Candesartan bilaterally injected into the RVLM of hypertensive rats decreased MAP by −27 ± 2.7 mmHg (baseline 162 ± 7.1 mmHg, *P* < .05, *n* = 5; Figure 2(A)). The fall in BP was preceded by a short lasting increase in this parameter, and the decrease after that was slow to develop (onset, 2.4 ± 0.9; peak, 9.4 ± 2.8 min) and lasted for approximately 30 minutes. In contrast, in control rats, the same injection had no significant effect on MAP (118 ± 4 to 111 ± 10 mmHg, *n* = 5), as shown in Figure 2(B).

### 3.3. Effects of 5-HT_1/2_ Receptor Antagonist Microinjection into the RVLM of Hypertensive Rats

**Figure 3:**
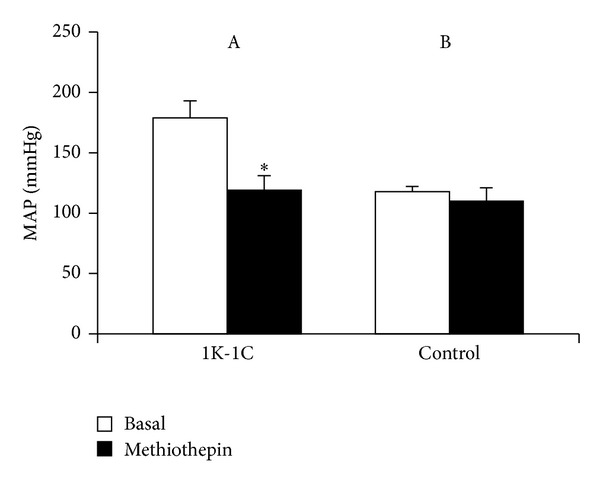
Values of mean arterial pressure (MAP) reached after bilateral microinjection of methiothepin (0.06 mol/L, 100 nL) into the RVLM in 1K-1C (*n* = 6) or control rats (*n* = 5). *P < .05 in relation to basal level.

The hypothesis examined in this series of experiments is whether the elevated blood pressure in 1K-1C rats is partially mediated by activation of 5-HT receptors within the RVLM. To address this hypothesis, 6 nmol of methiothepin was bilaterally injected into the RVLM of hypertensive or control rats. In the 1K-1C rats receiving bilateral injections of methiothepin (6 nmol) into the RVLM decreased MAP by 60 ± 14 mmHg (basal 179 ± 13 mmHg, *P* < .05, *n* = 6). The reduction was immediate and the latency to peak was in 12 ± 4 minutes.  Figure 3 shows the effects of methiothepin in control or 1K-1C rats. In control rats, methiothepin into the RVLM did not cause any change in blood pressure (from 118 ± 4 to 110 ± 12 mmHg, *n* = 5) or heart rate (from 436 ± 19 to 455 ± 10 bpm, *n* = 5).

## 4. Discussion

It is well established that the RVLM contains neurons involved in the tonic and reflex regulation of cardiovascular system and sympathetic drive and that an increase in RVLM activity could be an important mechanism in the pathogenesis of experimental hypertension. The major finding of the present study is that injection of AT_1_ Ang II or 5-HT receptor antagonists into the RVLM produced a significant decrease in MAP in 1K-1C rats; in contrast, in normotensive rats these treatments did not change MAP. Furthermore, the glutamatergic blockade within the RVLM did not change MAP or HR in both 1K-1C and control rats.

### 4.1. Role of Glutamatergic Transmission within the RVLM

Previous study from our laboratory demonstrated that in renovascular hypertensive rats (2 K-1C), the glutamatergic synapses within RVLM are tonically active, since injection of the broad-spectrum ionotropic glutamate receptor antagonist, kynurenic acid, into the RVLM causes a long lasting fall in blood pressure [5]. The role of tonic glutamatergic synapses within RVLM in the maintenance of hypertension was demonstrated not only in the Goldblatt model [5] but also in spontaneously hypertensive (SHR) and Dahl salt-sensitive rats [6, 9]. However, in the present study kynurenic acid microinjection into the RVLM in 1K-1C did not cause any change in BP, suggesting that there is no tonic glutamatergic action on RVLM in the 1K-1C model. However, glutamate microinjection into the RVLM caused larger response in 1K-1C than in control rats. A larger response to glutamate into the RVLM was also observed in SHR [12] and in 2K-1C hypertensive rats [5]. Nevertheless, in Dahl salt-sensitive rats the response to glutamate was not different from that in control rats [13], suggesting that the response to exogenous glutamate into the RVLM can be differential according to the hypertension model.

The importance of EAA drive to RVLM in the hypertension was also reported in acute experiments, in a model of mechanical compression of the RVLM that causes an increase in BP and sympathetic outflow. The pressor response to compression was inhibited after microinjection of Kyn into the RVLM [14]. Taken together, these results suggest that an increase in glutamatergic activity within RVLM is an important mechanism to increase BP, acutely or chronically, in different experimental models of hypertension.

### 4.2. Role of Angiotensinergic Transmission within the RVLM

We have shown that AT1 Ang II receptors antagonists into the RVLM have little effects of mean arterial pressure or sympathetic nerve activity of normotensive rats [10, 15–17]; however, they do decrease MAP and sympathetic activity in SHR and in Dahl salt-sensitive rats [9, 18] and in the present study using 1K-1C rats. The question that arises is as follows: which are the sources of the tonic angiotensinergic inputs to RVLM in hypertensive rats?

One possible source of angiotensinergic drive to RVLM is the hypothalamic paraventricular nucleus (PVN), which apparently is an important component in the central pathways mediating sustained increases in BP and sympathetic nerve activity in response to an increase in circulating Ang II. It is well known that the PVN receives inputs from angiotensinergic sensitive neurons located in the subfornical organ; therefore the circulating Ang II can act in this pathway increasing BP and sympathetic activity [19]. In addition, it was shown that the PVN is important to maintain elevated BP and sympathetic activity in experimental hypertension such as in the SHR and Dahl salt-sensitive rats [20, 21]. Finally, sympathoactivation in response to PVN activation is in part mediated by an activation of AT_1_ receptors within the RVLM. Tagawa and Dampney showed that the increase in MAP and sympathetic activation mediated by disinhibition of the PVN was significantly reduced by previous injection of losartan within the RVLM [10]. Therefore, it is possible that an increase in angiotensinergic drive from PVN to RVLM occurs in 1K-1C and may be involved in the maintenance of high BP. This mechanism was proposed to explain the high BP in Dahl-salt rats [18], SHR [20], and in chronic renal-wrap hypertension [22]. Alternatively, it is possible that Ang II acts in the brain from a nonneuronal origin; the peptide may be formed from angiotensinogen in the extracellular fluid and then reaches its receptors via diffusion [23].

### 4.3. Possible Role of 5-HT in 1K-1C Hypertension

Interestingly, not only the AT_1_ Ang II receptor drives RVLM in 1K-1C but also there is an important tonic excitatory influence mediated by serotonin (5-HT). In the present study, bilateral blockade of 5-HT receptors (mainly 5-HT_1_ and 5-HT_2_) within the RVLM causes profound fall in MAP. Central serotonergic pathways are known to innervate areas involved in cardiovascular regulation and 5-HT acts through two major receptors subtypes, 5-HT_1A_ and 5-HT_2_. Activation of the former induces sympathoinhibition while the latter sympathoexcitation [11]. In the present study we used a broad spectrum 5-HT antagonist–methiothepin; therefore, it is not possible to discriminate which 5-HT receptor is tonic driving the RVLM neurons. However, it is possible that the depressor response is mediated by blocking 5-HT_2_ receptors within the RVLM.

The importance of 5-HT_2_ in hypertension is illustrated by the fact that ketanserin, a selective 5-HT_2_ receptor antagonist, when centrally administrated causes central sympathoinhibition [24]. When the 5-HT_2_ receptor agonist DOI is administrated i.c.v. there is a rise in BP and sympathetic activation [24]. More importantly is the fact that the major central site of action of 5HT_2_ is the RVLM, since activation of these receptors in this region causes a rise in BP and sympathetic activation [25, 26]. However, a possible action of 5-HT systems in anterior region to the brain cannot be excluded, as the PVN and anterior hypothalamus (AH) are innervated by 5-HT neurons arising from medial and dorsal raphé nuclei. The increase in BP in response to raphé nuclei stimulation is in part mediated by 5-HT ascending projections from raphé to the PVN and AH. The pressor response evoked by nucleus raphé obscurus stimulation can be significantly reduced by sodium pentobarbital injection into RVLM [27]. Therefore, it is possible that activation of 5-HT receptors not only in the RVLM but also in other brain regions may be also involved in maintaining the elevated BP 1K-1C rats.

### 4.4. Limitations of the Present Study

The major limitation of the present study is that it is not possible to discriminate which 5-HT receptor is tonic driving the RVLM in the 1K-1C rats as well as the source of 5-HT projections and the interaction between AT1 and 5-HT receptors. Only with new experiments we will be able to address such issues.

### 4.5. Conclusions

The present study shows that blockade of AT_1_ or 5-HT receptors within the RVLM of 1K-1C rats, but not in normotensive control animals, decreases blood pressure. Additionally, apparently there is no tonic action of glutamate within the RVLM in this model. The difference in the role of RVLM in the control of arterial pressure in Goldblatt model and normotensive rats supports the hypothesis that changes in neurotransmission within the RVLM participate in the maintenance of high blood pressure in renovascular hypertension.

As stated before, the RVLM contains neurons involved in the tonic and reflex regulation of cardiovascular system and sympathetic drive. The present data suggest that changes in neurotransmission within the RVLM neurons in hypertensive rats with an increase in the angiotensinergic and serotonergic actions in this region contribute to the pathogenesis of renovascular hypertension. Understanding the mechanisms of neurotransmission alterations within premotor neurons in hypertension can improve our understanding on the mechanisms of sympathetic activation in this condition.
